# Risk-Based Care: Let's Think Outside the Box

**DOI:** 10.3389/fmed.2021.535244

**Published:** 2021-02-25

**Authors:** James Geoffrey Chase, Geoffrey M. Shaw, Jean-Charles Preiser, Jennifer L. Knopp, Thomas Desaive

**Affiliations:** ^1^Centre for Bioengineering, Mechanical Engineering, University of Canterbury, Christchurch, New Zealand; ^2^Department of Intensive Care, Christchurch Hospital, University of Otago Christchurch School of Medicine, Christchurch, New Zealand; ^3^Department of Intensive Care, Erasme University Hospital, Brussels, Belgium; ^4^GIGA In Silico Medicine, University of Liege, Liege, Belgium

**Keywords:** decision-making, risk, cost-benefit analysis, risk-based protocol, risk-based assessment

## Introduction

For intensive care medicine, significant advances in care have been limited in recent years. A wealth of randomized clinical trials and a wide range of new protocols have yielded a wealth of understanding but have not created the next-generation care hoped for. As a result, a “less is more” perspective is growing in the field. This opinion article looks to the field of economics for lessons in how we might consider changing perspective to develop and assess new approaches to care.

Current approaches to major forms of care in the intensive care unit (ICU) have stagnated, increasingly complex patients with multiple co-morbidities have resulted in increased use of resources, cost and risk of complications. Growing fear of iatrogenic complications has led to a “*less is more*” approach to new care (1–3). The major hurdle to overcome is the increase in negative outcomes, which seem to regularly arise with more aggressive approaches to care seen in a range of randomized clinical trials in the last two or more decades. This analysis takes an outside the box approach, with lessons from the field of Economics, to assess the situation and make some bold proposals, in the interest primarily of stimulating “creative tension” in the field as to considering the way forward.

## Cost-Benefit Analysis, So-Called Fat-Tailed Risk, and Medicine

A great deal of medical care treatment, perhaps particularly in the intensive care unit (ICU), can be seen as decision making under uncertainty. An economist might broadly define this process as assessing the potential value of the investment now vs. alternative outcome risks in the future, and discounting the “cost” of the outcome accordingly. Discounting adjusts the costs and outcomes of spending or interventions to account for investment benefits in delaying some or all of the cost of a treatment (4). The underlying issue is to determine how much to invest now – cost-benefit analysis (CBA) in simple. Thus, the question of what choice to make should be based on the relative “cost” of mistakes and their discounted cost.

However, the efficacy of CBA has been recently questioned based on its use in climate change analysis, challenged by Weitzman's “Dismal Theorem” (5). Weitzman used this theorem to show how traditional CBA could break down in issues like climate change, where avoiding a catastrophic future would demand an infinite investment now. By analogy, in the ICU, the ultimate cost to the patient is death, which would necessitate infinite investment in care using traditional CBA. However, such infinite investment, despite the actual high costs of critical care, is not typically made. This outcome demonstrates the fragility of CBA in the face of catastrophic or “fat tailed” risk, which occurs when catastrophic events can occur with non-negligible likelihood, no matter how remote the chance. More succinctly, the tail of the probability (of occurrence) distribution is “fat” and has non-negligible likelihood.

So-called “fat-tailed risk” arises from the formal definition of the Dismal Theorem noting society has an infinite or undefinable expected loss from very high consequence, but relatively very low-probability events. The infinite cost makes it impossible to calculate a present value to invest now to avoid it and thus no discount rate can apply to make a decision between options. The opposite, given human behavior, also arises, where low consequence, higher probability events in future are overly discounted and assumed to have a much lower or negligible impact and cost today due to a range of factors (6, 7).

More specifically, decision making for clinical staff and family is difficult, when we do not understand and cannot calculate the risks and thus value of a given treatment, when death or an unlikely event associated with increased mortality is a possible, albeit unlikely outcome. More importantly, the opposite case can also hold, where the risk of death or a negative outcome is assumed to be lower than is actually the case, and so actions may not be taken, the result of which are harmful to the patient. These “poor” choices or difficult decisions are a result of underlying uncertainty in patient response to care and thus in outcome, and in particular of our inability to quantify them to more optimally guide decision making and risk assessment in medical care, and in critical care in particular.

This difficult compromise can lead to poor choices, in both economics, but also as posited here, in medical decision-making – all complicated by the underlying uncertainty of what might occur in future, and the inability to properly assess underlying risk of both very unlikely, and more likely, events.

## Analogies in ICU Medicine

One clear, highly debated analogy in intensive care unit (ICU) medicine arises from the debate around glycemic control (GC) (8). Beyond potential benefit and the high difficulty in providing safe, effective GC for all patients (9–11), the risk of hypoglycemia—a potentially catastrophic event in terms of outcome risk of death (12–16)—is offset by the risk of permissive hyperglycemia from higher targets (17) and associated glycemic variability (18–21), which have much lower perceived relative risks, but are also associated with increased mortality. Thus, economically, there is failure to control or invest in GC given the so-called fat-tailed risk of hypoglycemia, where no current investment seems able to ameliorate the problem, and the opposite case also holding, where the risk of death due to permissive poor GC increases the risk of death due to underestimating the risk (over discounting).

This approach would provide an acceptable trade-off if there was no possibility to obtain good control, safe from hypoglycemia. Equally, the much lower (relative) and highly discounted perceived risk of hyperglycemia for ICU patients accepted in this trade-off clashes with the actual, higher relative risk observed (22–26). In contrast, emerging personalized, model-based GC approaches can provide safe, effective control with reduced hypoglycemia (1–2% of patients) (27–29), and emerging glucose sensing technologies offer further safety increases (30).

However, in short, there remains a tendency to “invest nothing,” and thus to minimal GC, to avoid the (now manageable) fat-tailed catastrophic risk of hypoglycemia. Concomitantly, there is thus also the resulting tendency to accept relatively permissive levels of hyperglycemia by under-valuing and over-discounting its negative effects and increased risk of death. These choices and resulting outcome match well-known and increasingly well-accepted economic observations and human behaviors.

However, the “Dismal Theorem” is an “impossibility theorem” and it does not tell you what you should do instead (31). Relevant to the position made here, Weitzman was quoted: “*We desperately need more information about what's going on in these tails. It's not the median values that are gonna kill us*” (32). While he said this concerning climate change, it could be equally applied to many risks and choices made in ICU medicine, not just GC. More directly, if we could quantify uncertainty and variability in response to care, we could appropriately assess the risk, and choose, economically speaking, the right discount rate to assess whether (or not) to “invest” in more aggressive care.

The trade-off between these types of risk can be found in many core ICU therapies. Mechanical ventilation has the trade-off between the sudden impact of barotrauma or volutrauma vs. the “slow” damage of increased inspired oxygen settings when setting PEEP or tidal volume (33). Thus, quantifying the risk, via new metrics or personalized models, would allow the proper assessment of patient response and thus whether (or not) to provide more aggressive ventilation settings, which if done incorrectly increase the risk of cost, length of stay, and mortality. Fluid resuscitation therapy faces a similar contradiction between providing more input to support circulatory and cardiac function, and the ongoing risk of the therapy itself to patient outcome (34, 35). Quantifying responsiveness to fluid resuscitation therapy is a “holy grail” of ICU research, as quantifying the response would reduce the risk and make it manageable. In all cases, difficulty arises from the neither knowing, quantifying nor managing the risk of outlying, harmful patient responses to care due to inter- and intra- patient variability.

## The Problem of Outliers, Variability and Risk

In general, many therapy approaches in randomized trials target median or mean behaviors, the central tendency of a cohort. However, they often fail to account for outliers or are unable to achieve these median goal targets for all or nearly all patients. Equally, those outlying events and patients can twist therapy targets due to their high risk of negative impact, such as hypoglycemia in GC. These outcomes suggest risk of behaviors for a therapy, rather than outcome, should be primary targets, particularly where fat-tailed risks can be quantified and managed.

Specifically, new approaches should seek to eliminate or directly manage outliers as they constitute the fat-tailed risk and are where both clinical and economical costs reside. This risk-based approach focuses less on achieving a target and more on minimizing any harm from care. It thus focuses on the knowing and managing the “tails” of the distribution of possible patient responses, rather than the central tendency or expected values, and in doing so might provide the means to develop therapies providing safe, effective care for virtually all patients. Overall, understanding and management of more immediate risks can allow better CBA and decision-making when it comes to evaluating interventions.

**Figure 1:**
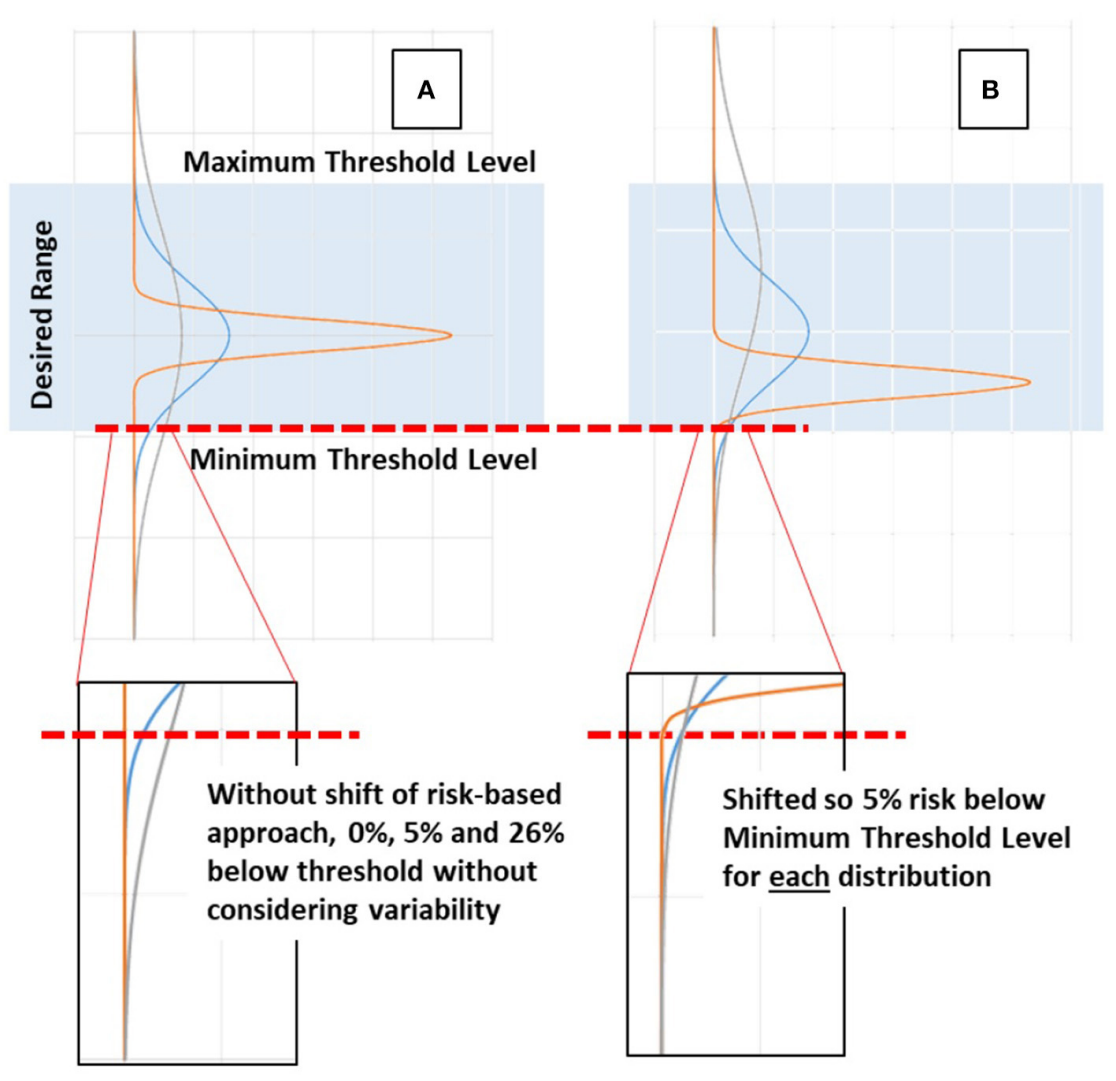
Schematic example of risk-based vs. typical approaches. **(A)** When distributions are unknown and median or central tendency values are targeted all distributions are centered together. A wide or fat-tailed distribution of response due to patient variability can create significant risk of being too low or too high compared to a desired range. **(B)** When the distribution and the tails in particular are known, then a set threshold of risk (5%) of being too low (or high) can be set and used to guide care, effectively shifting the distributions to a set lower bound in the example shown, and allowing the remainder of the distribution to fall where it may.

Figure 1 schematically shows how knowledge of potential variability in patient response can be used in a risk-based approach. When the distribution is unknown the response can be more or less variable, creating undesirable risk of a patient outcome response being too low (or too high) if the distribution is fat-tailed or wide. However, if the distribution is known, then care choices can be predicated on this knowledge, allowing a set threshold (5% in Figure 1) of risk in response to care. The analogy in the glycemic control case would be blood glucose below 4.0 mmol/L (72 mg/dL), associated with increasing risk.

Given the focus on uncertainty in Weitzman's work and in CBA in general, as applied economically, there is significant strong analogy to the practice of medicine. Using GC as a “straw man” it is particularly applicable to the design and evaluation of new therapy approaches, where randomized trials are often confounded by the complexity, multiple factors, and uncertainty governing major outcomes in ICU patients and thus fail to deliver clear direction to the field (2, 3). Thus, the ability to quantify and directly manage patient variability and risk in care would provide a new way of approaching not only care, but how it is developed and evaluated.

## Determining Variability and Managing Risk

Determining the distribution of risk or patient variability can be done using data, which is more and more available, both directly in statistical or stochastic models (36–38) or via a range of emerging artificial intelligence and/or deep learning methods (39). In fact, managing patient variability is an emerging field reflected in a range of recent research. Specifically in areas like new drug development methods, assessing differences between in response to care due to differences in race or sex, the use of a range of “omics” (e.g., proteomics, genomics) to better classify and target care, and in the broader use of “big data” and machine learning to everything from personalizing care to early warning alarms and other applications.

All of these approaches are emerging means to better classify patients and their potential needs, risks and variable responses (to care), sharing the common trait of bringing risk management into dosing and care. Doing so inherently creates increasingly personalized protocols. The act of personalizing care, whether done at the bedside or via algorithms and technology, is about managing patient-specific behaviors and responses to care outside the mean, median or central tendency, otherwise there would be no point to the effort. It is thus about giving all patients the best opportunity to respond like the expected target patient – inherently then, it is about risk reduction.

Once potential or actual variability and its risk is quantified in some way, it can be directly managed in care. Currently, only one glycemic control protocol takes such an approach in critical care, using a direct model-based approach to cohort variability to minimize hypoglycemia and provide consistent care across a range of cohorts (27). It shows the potential of this type of approach, vs. a targeted glycemia approach, and as data increases, greater resolution and personalization should be realized.

Hence, it is important to quantify variability for use in managing patient care to reduce the risk of outlier responses and to thus create ever narrower ranges of overall patient response to care. However, risk reduction is often a secondary outcome at best in many studies, with the primary focus being performance to desired clinical outcomes. If the future of ICU care is on personalizing care, and thus on directly managing and reducing risk, then it might be time to consider risk quantification, management and reduction as the primary goal in developing new approaches to care.

Surely if the risk of care can be managed and reduced, particularly in current care, then the increased personalization of care might help overcome a current stagnation in improving outcomes (and cost).

## A Bold Proposition?

As a result, this opinion and commentary puts forward two main propositions:

- Greater emphasis should be put on risk-based dosing, rather than on target-based dosing to minimize the likelihood of both fat-tailed and perceived lesser risks. In GC, dosing on risk to simultaneously minimize the relative risks of both hypoglycemia and permissive hyperglycemia. In short, dosing to minimize outliers rather than achieve a desired specific target or outcome.
- There needs to be far greater weight in clinical research put on evidence quantifying relative risks of choices in core ICU therapies, where large randomized trials on overall outcomes struggle given all the other uncertainties and complexity in the ICU patient (3).

More succinctly, when developing and evaluating protocols or approaches, like policy levers and choices in economics, risk-based and optimized decision making should be of concern over targeted median values and outcome focused approaches. This risk-based approach is not today's care, but should be tomorrow's.

## Discussion

The field of intensive care medicine faces significant challenges from increasing demographics and complexity of patients. However, it has seen far less significant advances in care compared to the level and intensity of research directed at the core problems it faces in care. This outside the box analysis examines the issue and through a set of “bold proposals” suggests the need for much greater quantification and direct management of risk in clinical care, treating outliers rather than seeking targets for the “middle.” It thus suggests re-examining how clinical evidence is viewed and redesigning how clinical trial goals are designed in an effort to address issues with variability and risk, which are currently under-served or ignored.

## Author Contributions

JC developed the idea and led writing. GS, J-CP, JK, and TD provided additional input and contributions to the development and writing. All authors contributed to the article and approved the submitted version.

## Conflict of Interest

The authors declare that the research was conducted in the absence of any commercial or financial relationships that could be construed as a potential conflict of interest.
